# Computational modeling of protein mutant stability: analysis and optimization of statistical potentials and structural features reveal insights into prediction model development

**DOI:** 10.1186/1472-6807-7-54

**Published:** 2007-08-16

**Authors:** Vijaya Parthiban, M Michael Gromiha, Madenhalli Abhinandan, Dietmar Schomburg

**Affiliations:** 1 Cologne University Bioinformatics Center, International Max Planck Research School, Cologne, Germany; 2 Computational Biology Research Center, National Institute of Advanced Industrial Science and Technology, Japan

## Abstract

**Background:**

Understanding and predicting protein stability upon point mutations has wide-spread importance in molecular biology. Several prediction models have been developed in the past with various algorithms. Statistical potentials are one of the widely used algorithms for the prediction of changes in stability upon point mutations. Although the methods provide flexibility and the capability to develop an accurate and reliable prediction model, it can be achieved only by the right selection of the structural factors and optimization of their parameters for the statistical potentials. In this work, we have selected five atom classification systems and compared their efficiency for the development of amino acid atom potentials. Additionally, torsion angle potentials have been optimized to include the orientation of amino acids in such a way that altered backbone conformation in different secondary structural regions can be included for the prediction model. This study also elaborates the importance of classifying the mutations according to their solvent accessibility and secondary structure specificity. The prediction efficiency has been calculated individually for the mutations in different secondary structural regions and compared.

**Results:**

Results show that, in addition to using an advanced atom description, stepwise regression and selection of atoms are necessary to avoid the redundancy in atom distribution and improve the reliability of the prediction model validation. Comparing to other atom classification models, Melo-Feytmans model shows better prediction efficiency by giving a high correlation of 0.85 between experimental and theoretical ΔΔG with 84.06% of the mutations correctly predicted out of 1538 mutations. The theoretical ΔΔG values for the mutations in partially buried *β*-strands generated by the structural training dataset from PISCES gave a correlation of 0.84 without performing the Gaussian apodization of the torsion angle distribution. After the Gaussian apodization, the correlation increased to 0.92 and prediction accuracy increased from 80% to 88.89% respectively.

**Conclusion:**

These findings were useful for the optimization of the Melo-Feytmans atom classification system and implementing them to develop the statistical potentials. It was also significant that the prediction efficiency of mutations in the partially buried *β*-strands improves with the help of Gaussian apodization of the torsion angle distribution. All these comparisons and optimization techniques demonstrate their advantages as well as the restrictions for the development of the prediction model. These findings will be quite helpful not only for the protein stability prediction, but also for various structure solutions in future.

## Background

Prediction of protein stability from structure is closely related to the prediction of structure from sequence, protein-protein and protein-ligand interactions [1], protein folding landscapes [2,3] and structure-function relationships [4]. Hence, it shares similar benefits and problems encountered by several methods available for those predictions. Knowledge-based potentials have been used in many aspects of protein structure solutions [5-10]. Hence, the prediction methods compared and optimized in this work are directly applicable for many such disciplines.

Several atom classification models [11-14] are available for the prediction of protein structure and stability with variable amount of success rates. There were also attempts to evaluate them [13] in order to select the one with the best definition suitable for a specific purpose. In this work, we have selected five classification models and compared them for their maximum efficiency in predicting protein stability. The atomic level organization of potentials for analyzing the radial distribution exhibits coverage of local and non-local interactions, and hence offers high accuracy for predictions [6]. As we described in our previous work [7], the energy functions are predominantly derived from the mean force potentials based on inverse Boltzmann's principle. Statistical methods were used to construct the prediction equation and eliminate the redundancy in the distribution of similar atoms in the atom model. This is especially important to ensure the reliability of prediction in the validation tests.

In our previous work [7], we evaluated the effect of torsion angle potentials and showed that their inclusion in the prediction increases the prediction accuracy. In this study, we have compared the torsion angle potentials with and without the Gaussian apodization in order to identify the mutations that adapt altered backbone torsion angles. Further, we have elaborated the work on the classification of mutations based on their solvent accessibility and secondary structure for improving the prediction efficiency and observed intuitive results in the development of prediction model. In our previous work, we compared the overall prediction efficiency of multiple prediction algorithms and evaluated the improvements of our own method. In general, some of the algorithms [15,16] were comparatively old and suffer from inadequacy due to lack of mutations. Few methods [17] and their improved versions [18,19] used a bigger dataset, but they used redundant mutation data which lead to unreliable prediction of protein stability. Cheng *et al*. [20] used SVMs and reported an accuracy of 84%. The current work differs by analysing the prediction efficiency independently for the mutations that are available in different regions of secondary structures and solvent accessibility. Additionally, five different atom classification models were taken and their prediction efficiencies were analysed. Results show that Melo-Feytmans model [21] offers better prediction efficiency compared to other atom classification models. Prediction efficiency of mutations in the partially buried *β*-strands improves with the help of Gaussian apodization of the torsion angle distribution. This leads to the conclusion that partially buried *β*-strands adapt altered backbone torsion angles in protein mutants.

## Methods

### Atom Classification Models

For the development of the protein stability prediction model, we selected five different atom classification methods for comparison. The first model classifies amino acid heavy atoms into 5 basic types: aliphatic and aromatic carbons, nitrogen, oxygen and sulphur. This is one of the simplest possible definitions for the amino acid atoms that can be used in a prediction model. For the second definition, we used 20 amino acid C*α *atoms as the representatives of amino acids. Additionally, we used 3 advanced atom models namely Li-Nussinov [11], SATIS [14] and Melo-Feytmans [12,21] model. Li and Nussinov classified the amino acid heavy atoms into 24 different types that reflect the maximum variation in VDW contact radii. The classification criterion was based on the possible number of hydrogen bonds and/or covalent bonds that can be formed between two heavy atoms. The SATIS method classifies the heavy atoms into 28 types according to their covalent connectivity. The Melo-Feytmans model classifies the heavy atoms into 40 types according to their location (backbone/side-chain), connectivity and chemical nature. The definitions used by these classification methods are closely linked to protein structure and stability features and a comparison of these methods aids in the selection of an optimal model for the amino acid-atom potentials. Torsion angle potentials were retained without any change for this comparison. Pearson's correlation coefficient between the predicted and experimental ΔΔG values for 1538 mutations taken from thermal denaturation experiments was used as the quality criterion to compare these models. These mutations were mainly taken from the Protherm web database [22] and the literature [7]. Additionally, prediction accuracy of mutations (correctly predicted as stabilizing or destabilizing) was observed together with the correlation coefficient.

### Effect of Gaussian Apodization and Torsion Angle Potentials

As we described in our previous work [7], torsion angle potentials (*f(φ, ψ)*) were developed using the main backbone torsion angles *φ *and *ψ*.

f(φ,ψ)=12πσ2⋅A(φ,ψ)
 MathType@MTEF@5@5@+=feaafiart1ev1aaatCvAUfKttLearuWrP9MDH5MBPbIqV92AaeXatLxBI9gBaebbnrfifHhDYfgasaacH8akY=wiFfYdH8Gipec8Eeeu0xXdbba9frFj0=OqFfea0dXdd9vqai=hGuQ8kuc9pgc9s8qqaq=dirpe0xb9q8qiLsFr0=vr0=vr0dc8meaabaqaciaacaGaaeqabaqabeGadaaakeaacqWGMbGzcqGGOaakiiGacqWFgpGzcqGGSaalcqWFipqEcqGGPaqkcqGH9aqpdaWcaaqaaiabigdaXaqaaiabikdaYiab=b8aWjab=n8aZnaaCaaaleqabaGaeGOmaidaaaaakiabgwSixlabdgeabjabcIcaOiab=z8aMjabcYcaSiab=H8a5jabcMcaPaaa@4517@

Here, *σ *is the standard deviation. The torsion angle distribution was normalized with a standard procedure using the circular apodization function (*A(φ, ψ)*) for *φ *and *ψ *having the bivariate normal distribution. Apodization of torsion angle distribution enables the mutants to adapt slightly altered backbone torsion angle combinations. This improves the accuracy in predictions by assigning favorable energy values to the neighboring values of frequently encountered torsion angle (*φ*-*ψ*) combinations. Apodization is carried out by the Gaussian function although other variants (Blackman, Hamming or Connes apodization functions [23]) may contribute similarly to the torsion angle distribution:

A(φ,ψ)=e−((φ−μφ)2+(ψ−μψ)22σ2)
 MathType@MTEF@5@5@+=feaafiart1ev1aaatCvAUfKttLearuWrP9MDH5MBPbIqV92AaeXatLxBI9gBaebbnrfifHhDYfgasaacH8akY=wiFfYdH8Gipec8Eeeu0xXdbba9frFj0=OqFfea0dXdd9vqai=hGuQ8kuc9pgc9s8qqaq=dirpe0xb9q8qiLsFr0=vr0=vr0dc8meaabaqaciaacaGaaeqabaqabeGadaaakeaacqWGbbqqcqGGOaakiiGacqWFgpGzcqGGSaalcqWFipqEcqGGPaqkcqGH9aqpcqWGLbqzdaahaaWcbeqaaiabgkHiTmaabmaabaWaaSaaaeaacqGGOaakcqWFgpGzcqGHsislcqWF8oqBdaWgaaadbaGae8NXdygabeaaliabcMcaPmaaCaaameqabaGaeGOmaidaaSGaey4kaSIaeiikaGIae8hYdKNaeyOeI0Iae8hVd02aaSbaaWqaaiab=H8a5bqabaWccqGGPaqkdaahaaadbeqaaiabikdaYaaaaSqaaiabikdaYiab=n8aZnaaCaaameqabaGaeGOmaidaaaaaaSGaayjkaiaawMcaaaaaaaa@4FFB@

Here, *μ*_*φ *_and *μ*_*ψ *_are the degrees of torsion angle alterations. When large numbers of protein structures are used from the structural training datasets, the torsion angle distribution is observed accurately having sufficient counts for many torsion angle combinations. In order to achieve the best prediction results, the maximum values of *μ*_*φ *_and *μ*_*ψ *_(Equation 2) were optimized so that the girth around the *φ*-*ψ *peaks is adjusted accordingly to produce the best prediction efficiency with higher correlation with experimental ΔΔG.

In order to ensure that the effect of the Gaussian function is not influenced by varying number of torsion angle combinations that may exist in different structural training datasets, three different datasets were taken for the development of torsion angle potentials: a non-redundant dataset with 4024 protein chains derived from PISCES [24], a non-redundant dataset (40% sequence identity cut-off) from SCOP-ASTRAL [25] and the Top500 [26] dataset which has been used previously as representative torsion angle dataset by others.

### Regression Methods

Multiple regression method with forward stepwise selection was used to fit the theoretically calculated energy values from atom distribution with the experimental ΔΔG. Here, the atoms were fit with experimental data using dynamic regression coefficients. These regression coefficients were calculated for all the atoms and torsion angle potential, by regressing the calculated stabilisation energy values with the experimental ΔΔG. Prediction algorithms were developed individually for the five atom models and their results were compared. The equations were separately calculated for the mutations classified into various structural regions. The optimization process was also carried out for different regions using the same algorithm. This enables us to compare the prediction ability of the selected atom models across the structural regions.

As we described in our previous publication, we have used the correlation coefficient between the predicted and experimental ΔΔG of 1538 point mutations derived from the thermal denaturation experiments. The correlation coefficients from five atom classification models across different structural regions were compared. The prediction model with higher correlation between the experimental and predicted ΔΔG is considered a better model.

### Cross Validation Tests

Three independent cross-validation tests were used to prove that the statistical potentials from atom types and torsion angles can be used to develop an efficient prediction model: Split-sample, jack-knife and k-fold cross-validation tests. For the split-sample validation, the total mutation dataset was broken into two representative datasets. It was made sure that mutations from different structural regions were equally populated in these two datasets every time. The prediction equation was developed from one dataset and the same was applied to the other. For the jack-knife test, every single mutation was individually tested with the equation developed from the remaining mutations. For k-fold cross validation, 3-, 4- and 5-folds were considered where the mutation dataset was broken into 3, 4 and 5 representative datasets and tested against each other.

## Results and discussion

**Figure 1:**
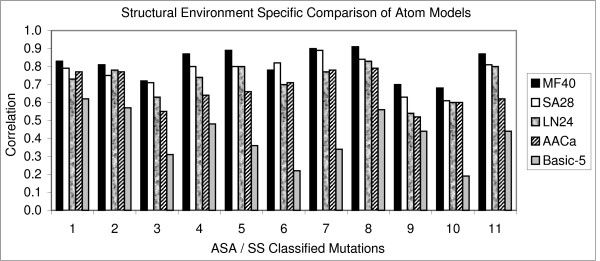
**Prediction efficiency of atom models – correlation **for predicting thermal ΔΔG values. Structural environment specific comparison of 5 different atom classification models used in the prediction of changes in protein stability. Correlation (1) and prediction accuracy (2) for predicting thermal ΔΔG values. Prediction efficiencies in 11 different structural regions were compared using CL11 classification method [7]. This method classifies mutations into 11 structural regions [helices (0–2, 2–30, 30–60, 60+), strands (0–5, 5–35, 35+) and others (0–10, 10–42, 42–67, 67+)]. MF40, SA28 and LN24 stand for the Melo-Feytmans, SATIS and Li-Nussinov atom models respectively.

**Figure 2:**
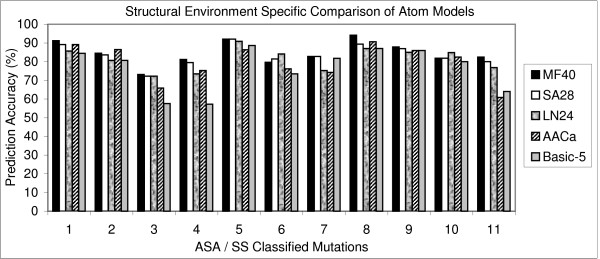
**Prediction efficiency of atom models – prediction accuracy **for predicting thermal ΔΔG values. Structural environment specific comparison of 5 different atom classification models used in the prediction of changes in protein stability. Correlation (1) and prediction accuracy (2) for predicting thermal ΔΔG values. Prediction efficiencies in 11 different structural regions were compared using CL11 classification method [7]. This method classifies mutations into 11 structural regions [helices (0–2, 2–30, 30–60, 60+), strands (0–5, 5–35, 35+) and others (0–10, 10–42, 42–67, 67+)]. MF40, SA28 and LN24 stand for the Melo-Feytmans, SATIS and Li-Nussinov atom models respectively.

The correlation coefficient (Fig. 1) and prediction accuracy (Fig. 2) of all the atom models were derived separately and compared. The Melo-Feytmans model showed the best results among all the atom classification models followed by the SATIS atom model (Fig. 1). The former showed a correlation of 0.85 with 84.06% of the mutations correctly predicted out of 1538 mutations. The SATIS model showed a slightly reduced correlation of 0.82 with 82.96% of mutations correctly predicted. Correlation and prediction accuracy gradually reduced for other atom models that had less number of atom types. It can be concluded that the size of the atom model is directly proportional to the increase in correlation. This is due to an elaborate definition of protein environment of any bigger atom model. However, a statistical problem of over-fitting of the atom types cannot be averted for a bigger atom model definition, since the regression method has too many parameters (predictors or atoms) offered by a bigger atom model. An absurd model may fit perfectly, if the model has enough complexity by comparison to the amount of available mutation data. In addition, it may end up with multiple prediction models that are significantly different, yet offer equally good correlation between experimental and predicted ΔΔG values due to over-fitting of atom parameters. Moreover, the prediction model requires the simplest possible atom classification system to ensure its reliability and to pass multiple validation tests. Hence, the solution for this problem was to use a smaller atom classification model, either statistically reduced or a model being small by default. So, the selection of specific atom types using their statistical significance, combined with stepwise linear regression was carried out to reduce the number of atom types. We proved in our previous publication that these statistical models provided good correlation between predicted and experimental ΔΔG, where the reduced Melo-Feytmans atom classification system performed better than other atom models [7].

To get further insight on the capacity of these atom models, protein environment specific prediction efficiency was also analyzed. The prediction algorithm using these atom models showed a good correlation for the mutations in the buried and exposed region compared to partially buried region of the protein. For the Melo-Feytmans model, a correlation of 0.84 was observed for the mutations in the buried helix regions (Fig. 1: ASA/SS classified structural region 1). The correlation slightly decreased to 0.81 and 0.71 for the mutations in the partially buried region of protein (Fig. 1: ASA/SS classified structural regions 2 and 3 respectively). However, the correlation increased in the exposed region of the helices (Fig. 1: structural region 4). Similar effect was observed for all the other atom models in almost all structural regions.

A decrease in the correlation between experimental and theoretical ΔΔG was observed in partially buried regions of the protein for all the models (Fig. 1). It can be clearly seen that all atom models predict mutations in buried and exposed regions very well compared to the partially buried region. Due to high conservation of atom distribution in compact structural regions of proteins and the atom potentials' ability to include hydrophobic interactions, the prediction model showed consistently good results in buried structural regions. In the partially buried helix residues, the prediction model showed slightly decreased correlation and prediction accuracy because it could not assess the stabilizing effect of some of these residues, since the parameters from atom potentials were not as effective as in the buried regions.

Parallel and anti-parallel *β*-strands are different in their hydrogen bonding patterns. They were not distinguished because a smaller number of mutations were found in strands and a dedicated prediction model for each of them would not be feasible. In the mutation dataset, there were more stabilizing mutations in partially buried turns and coils. These residues tend to achieve stability due to the formation of favorable new interactions. These interactions are newly established due to the flexibility of side chains in the partially buried region.

The mutations in the exposed region were also found to have higher correlation with the experimental ΔΔG. These mutations are influenced by many unknown medium and long range interactions. Usually, statistical potentials are better than empirical energy functions in assessing the unknown medium and long range interactions [27,28]. Exposed turns and coils are highly flexible regions in proteins which are mostly involved in initiating the folding-unfolding transition. Long range interactions play a significant role in all these cases [7]. Due to this reason, they mainly initiate the unfolding process, even as a result of slight changes in environmental conditions. Stabilizing and destabilizing mutations were equal in number and easily distinguished in this region. Thus, the statistical potentials combined with classification of structural regions, are more effective in these structural regions and help us to overcome the previously reported problems [6] to develop an efficient prediction model.

The correlation for the mutations in partially buried strands (ASA/SS classified structural region 6 in fig. 1) for the Melo-Feytmans model was slightly lower (correlation coefficient = 0.78), compared to the correlation (0.82) observed for the SATIS model. This was the only case for better performance of a smaller atom model, which is due to the over-fitting of the data by the Melo-Feytmans model. This behavior further supports the necessity of dimensionality reduction techniques to optimize the size of atom models. We used multicolinearity diagnostics and stepwise regression to eliminate this over-fitting effect in all structural regions and showed a reliable prediction model earlier [7]. Prediction accuracy (%) was found to be similar to the observed correlation coefficient between predicted and experimental ΔΔG, although there were minor exceptions in some structural regions. However, correlation is given higher importance in such cases because a high correlation with ΔΔG always supports the majority of mutations to be correctly predicted as stabilizing or destabilizing, but it's not vice versa.

**Figure 3:**
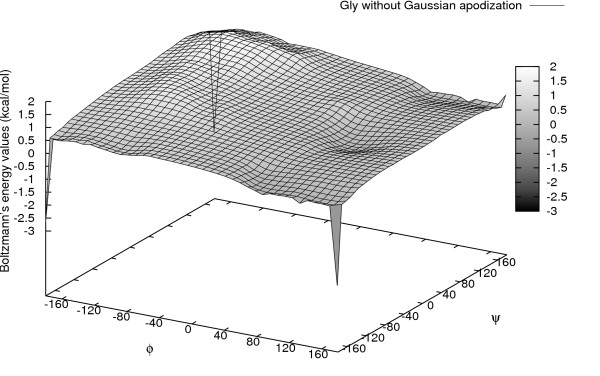
**Effects of Gaussian apodization of torsion angles – **energy values calculated before the Gaussian apodization. Boltzmann's energy distribution derived from torsion angles *φ *and *ψ *for the amino acid Gly. Corners of the distribution graphs are denoted with sharp legs/edges (shown up or down depending on general distribution data value). Figure 3 shows the energy values calculated before the Gaussian apodization.

**Figure 4:**
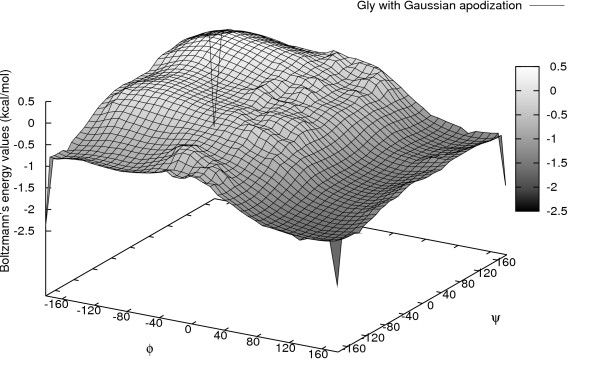
**Effects of Gaussian apodization of torsion angles – **energy values calculated after the Gaussian apodization. Boltzmann's energy distribution derived from torsion angles *φ *and *ψ *for the amino acid Gly. Corners of the distribution graphs are denoted with sharp legs/edges (shown up or down depending on general distribution data value). Figure 4 shows the energy values calculated after the Gaussian apodization.

Although we showed in our previous publication that the Gaussian function improved the overall prediction efficiency, it would be of particular interest to analyze and learn which secondary structural regions benefit from this function. Fig. 3 and Fig. 4 show the Boltzmann's energy values calculated from the torsion angle distribution of Gly before and after the Gaussian apodization respectively. Apodization was done for the maximum of 7 degrees in either direction for both the angles *φ *and *ψ*. This leaves us with 196 combinations from the *φ*-*ψ *distribution (Fig 4).

**Figure 5:**
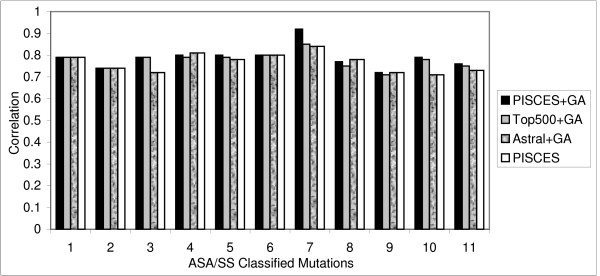
**Prediction efficiency of structural training datasets & Gaussian apodization – **Correlation for predicting mutations with thermal ΔΔG values. Comparison of structural training datasets to render torsion angle potentials. For the atom potentials, the PISCES dataset was used as structural training dataset. For the torsion angle potentials, 3 datasets were compared: PISCES, SCOP-ASTRAL and Top500. Correlation and prediction accuracy for predicting mutations with thermal ΔΔG values. PISCES-GA and PISCES denote respectively, the datasets used with and without Gaussian apodization.

**Figure 6:**
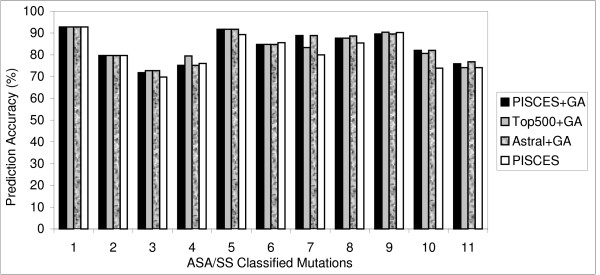
**Prediction efficiency of structural training datasets & Gaussian apodization – **Prediction accuracy for predicting mutations with thermal ΔΔG values. Comparison of structural training datasets to render torsion angle potentials. For the atom potentials, the PISCES dataset was used as structural training dataset. For the torsion angle potentials, 3 datasets were compared: PISCES, SCOP-ASTRAL and Top500. Correlation (5) and prediction accuracy (6) for predicting mutations with thermal ΔΔG values. PISCES-GA and PISCES denote respectively, the datasets used with and without Gaussian apodization.

Fig. 5 compares the correlation coefficient between predicted and experimental ΔΔG values across the secondary structural regions. Results show that, with respect to other structural regions, mutations in exposed *β*-strands are predicted more efficiently after applying the Gaussian apodization. The theoretical ΔΔG values for the mutations in partially buried *β*-strands generated by the structural training dataset from PISCES gave a correlation of 0.84 without performing the Gaussian apodization of the torsion angle distribution. After the Gaussian apodization, the correlation increased to 0.92 and prediction accuracy increased from 80% to 88.89% respectively. Since these mutations are not in the compact region, they have higher levels of flexibility and achieve a stabilizing conformation even after a significant change in backbone torsion angles *φ *and *ψ*. Comparing to other structural training datasets considered for the torsion angle potential, PISCES performed better in many of the secondary structural regions (Fig 5, 6). This study leads to further optimize the Gaussian function in future in such a way that every structural region will assume unique parameters (angle bins, maximum degrees of flexibility). For the model validation, we reported in our previous publication that the split-sample validation gave a correlation (standard error) of 0.87 (0.71 kcal/mol) for the training dataset, and 0.77 (0.95 kcal/mol) for the representative test dataset. Correctly predicted mutations for the training and test datasets were observed to be 84.6% and 81.6% respectively. For selected methods of three, four and five subsets of dataset breakage, the average correlation (standard error) was observed to be 0.75 (0.99 kcal/mol) with 81% of the mutations correctly predicted. This shows that the prediction model can be used for protein stability predictions. After removing 20 outliers observed during the above validation tests, jack-knife validation was carried out which gave a correlation coefficient of 0.70 (1.17 kcal/mol) with prediction efficiency of 77.84%. These validation tests were highly supportive for using the prediction algorithm for further analyses in the current work that include the comparison of atom classification models and the effect of Gaussian apodization.

## Conclusion

We have compared multiple atom classification models and optimized the final prediction model (Melo-Feytmans) using statistical methods and other structural features to predict protein stability changes upon point mutations. Moreover, we have also compared the prediction efficiency of the model in multiple secondary structural regions. We have also demonstrated the efficiency of the Gaussian apodization for the torsion angle potentials and simultaneously compared the different structural training datasets for the influence on the development of the torsion angle potentials. All these comparisons and optimization techniques demonstrate their advantages as well as the restrictions for the development of the prediction model. These findings will be helpful not only for the protein stability prediction, but also for various structure solutions in future.

## Authors' contributions

VP, MMG and DS conceived the study. VP developed the algorithm, carried out the computations and analyzed the results. MMG created the dataset and MA provided support in organizing the dataset and doing few analyses. VP prepared the manuscript and MMG revised the manuscript. DS contributed on various discussions and provided guidance in developing the algorithm. All authors read and approved the manuscript.

## References

[B1] Zhang C, Liu S, Zhu Q, Zhou Y (2005). A knowledge-based energy function for protein-ligand, protein-protein, and protein-DNA complexes. J Med Chem.

[B2] Fersht AR (1995). Characterizing transition states in protein folding: an essential step in the puzzle. Curr Opin Struct Biol.

[B3] Martinez JC, Pisabarro MT, Serrano L (1998). Obligatory steps in protein folding and the conformational diversity of the transition state. Nat Struct Biol.

[B4] Beadle BM, Shoichet BK (2002). Structural bases of stability-function tradeoffs in enzymes. J Mol Biol.

[B5] Hoppe C, Schomburg D (2005). Prediction of protein thermostability with a direction- and distance-dependent knowledge-based potential. Protein Sci.

[B6] Khatun J, Khare SD, Dokholyan NV (2004). Can contact potentials reliably predict stability of proteins?. J Mol Biol.

[B7] Parthiban V, Gromiha MM, Hoppe C, Schomburg D (2007). Structural analysis and prediction of protein mutant stability using distance and torsion potentials: role of secondary structure and solvent accessibility. Proteins.

[B8] Parthiban V, Gromiha MM, Schomburg D (2006). CUPSAT: prediction of protein stability upon point mutations. Nucleic Acids Res.

[B9] Samudrala R, Moult J (1998). An all-atom distance-dependent conditional probability discriminatory function for protein structure prediction. J Mol Biol.

[B10] Zhang C, Liu S, Zhou Y (2004). Accurate and efficient loop selections by the DFIRE-based all-atom statistical potential. Protein Sci.

[B11] Li AJ, Nussinov R (1998). A set of van der Waals and coulombic radii of protein atoms for molecular and solvent-accessible surface calculation, packing evaluation, and docking. Proteins.

[B12] Melo F, Feytmans E (1998). Assessing protein structures with a non-local atomic interaction energy. J Mol Biol.

[B13] Mintseris J, Weng Z (2004). Optimizing protein representations with information theory. Genome Inform.

[B14] Mitchell JBO, Alex A, Snarey M (1999). SATIS: Atom Typing from Chemical Connectivity. J Chem Inf Model.

[B15] Gilis D, Rooman M (2000). PoPMuSiC, an algorithm for predicting protein mutant stability changes: application to prion proteins. Protein engineering.

[B16] Guerois R, Nielsen JE, Serrano L (2002). Predicting changes in the stability of proteins and protein complexes: a study of more than 1000 mutations. J Mol Biol.

[B17] Capriotti E, Fariselli P, Casadio R (2004). A neural-network-based method for predicting protein stability changes upon single point mutations. Bioinformatics.

[B18] Capriotti E, Fariselli P, Calabrese R, Casadio R (2005). Predicting protein stability changes from sequences using support vector machines. Bioinformatics.

[B19] Capriotti E, Fariselli P, Casadio R (2005). I-Mutant2.0: predicting stability changes upon mutation from the protein sequence or structure. Nucleic Acids Res.

[B20] Cheng J, Randall A, Baldi P (2006). Prediction of protein stability changes for single-site mutations using support vector machines. Proteins.

[B21] Melo F, Feytmans E (1997). Novel knowledge-based mean force potential at atomic level. J Mol Biol.

[B22] Gromiha MM, An J, Kono H, Oobatake M, Uedaira H, Sarai A (1999). ProTherm: Thermodynamic Database for Proteins and Mutants. Nucleic Acids Res.

[B23] Weisstein EW "Apodization Function." From MathWorld – A Wolfram. http://mathworld.wolfram.com/ApodizationFunction.html.

[B24] Wang G, Dunbrack RL (2003). PISCES: a protein sequence culling server. Bioinformatics.

[B25] Chandonia JM, Hon G, Walker NS, Lo Conte L, Koehl P, Levitt M, Brenner SE (2004). The ASTRAL Compendium in 2004. Nucleic Acids Res.

[B26] Word JM, Lovell SC, LaBean TH, Taylor HC, Zalis ME, Presley BK, Richardson JS, Richardson DC (1999). Visualizing and quantifying molecular goodness-of-fit: small-probe contact dots with explicit hydrogen atoms. J Mol Biol.

[B27] Gromiha MM, Selvaraj S (2004). Inter-residue interactions in protein folding and stability. Prog Biophys Mol Biol.

[B28] Lazaridis T, Karplus M (2000). Effective energy functions for protein structure prediction. Curr Opin Struct Biol.

